# Development of Orally Administered γ-Tocotrienol (GT3) Nanoemulsion for Radioprotection

**DOI:** 10.3390/ijms18010028

**Published:** 2016-12-24

**Authors:** Grace A. Ledet, Shukla Biswas, Vidya P. Kumar, Richard A. Graves, Demaurian M. Mitchner, Taylor M. Parker, Levon A. Bostanian, Sanchita P. Ghosh, Tarun K. Mandal

**Affiliations:** 1 Center for Nanomedicine & Drug Delivery, College of Pharmacy, Xavier University of Louisiana, New Orleans, LA 70125, USA; grace.ledet@gmail.com (G.A.L.); rgraves@xula.edu (R.A.G.); dmitchne@xula.edu (D.M.M.); tparker4@xula.edu (T.M.P.); lbostani@xula.edu (L.A.B.); 2 Armed Forces Radiobiology Research Institute, Uniformed Services University of the Health Sciences, Bethesda, MD 20814, USA; shukla.biswas.ctr@usuhs.edu (S.B.); vidya.kumar.ctr@usuhs.edu (V.P.K.)

**Keywords:** γ-tocotrienol, GT3, nanoemulsion, dry emulsion, radioprotection, lyophilization

## Abstract

The purpose of this study was two-fold: (1) to formulate γ-tocotrienol (GT3) in a nanoemulsion formulation as a prophylactic orally administered radioprotective agent; and (2) to optimize the storage conditions to preserve the structural integrity of both the formulation and the compound. γ-tocotrienol was incorporated into a nanoemulsion and lyophilized with lactose. Ultra performance liquid chromatography–mass spectroscopy (UPLC–MS) was used to monitor the chemical stability of GT3 over time, the particle size and ζ potential, and scanning electron microscopy (SEM) were used to study the physical stability of the nanoemulsion. Radioprotective and toxicity studies were performed in mice. The liquid formulation exhibited GT3 degradation at all storage temperatures. Lyophilization, in the presence of lactose, significantly reduced GT3 degradation. Both the liquid and lyophilized nanoemulsions had stable particle size and ζ potential when stored at 4 °C. Toxicity studies of the nanoemulsion resulted in no observable toxicity in mice at an oral dose of 600 mg/kg GT3. The nano-formulated GT3 (300 mg/kg) demonstrated enhanced survival efficacy compared to GT3 alone (200 and 400 mg/kg) in CD2F1 mice exposed to total body gamma radiation. The optimal long-term storage of formulated GT3 is as a powder at −20 °C to preserve drug and formulation integrity. Formulation of GT3 as a nanoemulsion for oral delivery as a prophylactic radioprotectant shows promise and warrants further investigation.

## 1. Introduction

γ-tocotrienol (γ-tocotrienol, GT3) is one of the eight naturally-occurring isomers of vitamin E (sometimes collectively called tocols). The strong antioxidant properties, ease of absorption, and minimal toxicity of GT3 has sparked research into GT3 as a radioprotective agent [1,2,3]. GT3 has been shown to be superior to α-tocopherol, the most abundant vitamin E isoform, in reducing lethality after total-body irradiation (TBI) when given 24 h before cobalt-60 irradiation at 11 Gy [4]. GT3 protects against hematopoietic radiation toxicity by ameliorating intestinal radiation injury, enhancing intestinal recovery after TBI, and inhibiting hydroxyl-methyl-glutaryl-coenzyme A (HMG-CoA) reductase, which reduces vascular oxidative stress [5]. Indeed, GT3 has stronger inhibitory effects on HMG-CoA compared to other tocols [6,7] and has faster intestinal absorption than other tocols [8].

The development of radioprotective agents which can be used prior to exposure is critical for first responders within a radiation exposure field [4], and, particularly, oral administration has clinical relevance as an effective countermeasure for both first responders as well as for at-risk civilian populations in a nuclear accident [9]. While a single dose of GT3 (200 mg/kg) delivered subcutaneously 24 h prior to exposure can protect 100% of CD2F1 mice from radiation-induced injury and death [1,10], oral delivery for radioprotection has not been studied. As an oil extract at room temperature, GT3 can be successfully formulated as a nanoemulsion for oral delivery. Formulation of α-tocopherol has been proven to increase its radioprotective efficacy [11]. Nanoemulsions are kinetically stable emulsions with an internal droplet size in the nanometer range. While nanoemulsions can be stable for a prolonged period of time, their kinetic nature still makes them susceptible to creaming, coalescence, flocculation, and Ostwald ripening [12]. Additionally, the oil phase can be subjected to oxidation, and nanoemulsions can be very sensitive to storage temperature. Drying an emulsion is one alternative to overcome the disadvantages related to emulsion formulations and drug instability. The most common methods to prepare dry powder formulations are spray drying and freeze drying. Both of these methods are equally effective in drying nanoemulsion formulations, however the optimum choice depends on the economics of the process and the intended route of administration [13]. Freeze drying is preferred for heat sensitive and injectable materials, but the process is slow. In contrast, spray drying is faster and suitable for inhalable powder formulations, but the process uses high temperature gas which may be detrimental to some heat sensitive drugs [14]. The goal of a dry emulsion is to enable immediate recovery of the original oil-in-water (o/w) emulsion after rehydration. To preserve the emulsion following the removal of the aqueous phase of the emulsion, whether by freeze drying or by spray drying, a shell material must be incorporated into the formulation prior to drying. As the name implies, the role of the shell material is to encase the lipid droplets of the emulsion to prevent oil coalescence and oxidation. While compelling evidence of GT3’s radioprotective efficacy exists, the oral absorption of GT3 and other tocotrienols is incomplete, and the absolute bioavailability of GT3 is only around 9% [15]. Dry emulsions can increase the bioavailability and intestinal absorption of compounds [16,17].

The goal of this study was two-fold: (1) to formulate GT3 in a nanoemulsion (NE) formulation as an improved prophylactic orally administered radioprotective agent; and (2) to optimize the storage conditions of the formulation in order to preserve the structural integrity of both the formulation and the compound. Nanoemulsion formulations were prepared both in the liquid state and solid state. Stability studies for both formulations were conducted at 25, 4, and −20 °C for 24 weeks. Both the physical and chemical stability of the formulation and the drug were monitored. A 14-day acute toxicity study was performed following oral delivery of the nanoemulsion formulation with and without GT3. Finally, the radioprotective efficacy of the GT3 nanoemulsion was compared to that of unformulated GT3 following oral administration 24 h prior to radiation exposure.

## 2. Results

### 2.1. Physical Characterization of the Liquid and Lyophilized Nanoemulsion Formulations

**Figure 1:**
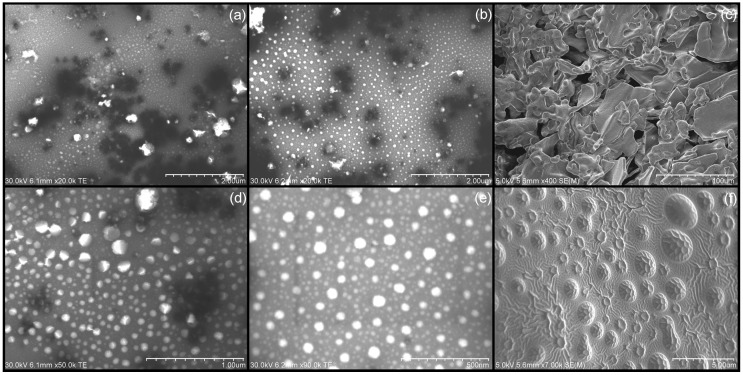
Scanning electron microscopy (SEM) micrographs of liquid γ-tocotrienol nanoemulsion (GT3 NE) (**a**) low magnification; (**d**) high magnification; reconstituted lyophilized GT3 NE (**b**) low magnification; (**e**) high magnification; and lyophilized GT3 NE (**c**) low magnification; (**f**) high magnification.

The liquid NE containing GT3 (liquid GT3 NE) had comparable physical characteristics to that of the lyophilized NE containing GT3 (lyophilized GT3 NE): median particle size of 130 to 150 nm, unimodal size distribution, and ζ potential of −30 to −40 mV. The predominant dissimilarity between the liquid GT3 NE and the lyophilized GT3 NE involved the polydispersity index of the formulations. While the median particle size and ζ potential were not statistically different following lyophilization of the GT3 NE, the polydispersity index of the reconstituted lyophilized GT3 NE was statistically different from that of the liquid GT3 NE (*p* = 0.002). The reconstituted lyophilized GT3 NE had a polydispersity index of 0.224 ± 0.021, and the liquid GT3 NE was measured at 0.099 ± 0.021. The morphology of the reconstituted lyophilized GT3 NE was comparable to the size and shape of the liquid GT3 NE (Figure 1). The lyophilized GT3 NE resulted in large, irregular flakes or plates without any distinctive macro-structure, but did result in a distinctive micro-structure (Figure 1f). The surface of the flakes or plates had a regular, truncated dome array. The liquid NE without GT3 (liquid blank NE) had a polydispersity index of 0.153 ± 0.044, median particle size of 94.7 ± 34.1 nm, and ζ potential of −28.76 ± 8.11 mV. Upon reconstitution, the lyophilized NE without GT3 (lyophilized blank NE) resulted in a nanoemulsion with a polydispersity of 0.218 ± 0.009, median particle size of 153.0 ± 27.2 nm, and a ζ potential of −30.00 ± 9.94 mV. No degradation of GT3 was detected in any of the samples immediately following formulation. While no distinction could be made between the liquid GT3 NE and blank NE and between the lyophilized GT3 NE and blank NE, the integrity of GT3 in the formulations did distinguish the different formulations from one another when studied after storage at different temperatures.

### 2.2. Stability of the Nanoemulsion Formulations

**Figure 2:**
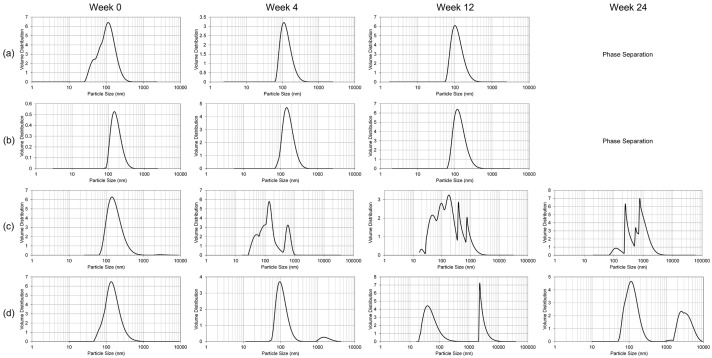
Particle size distribution over time for (**a**) liquid blank NE; (**b**) liquid GT3 NE; (**c**) lyophilized blank NE; and (**d**) lyophilized GT3 NE stored at 25 °C.

**Figure 3:**
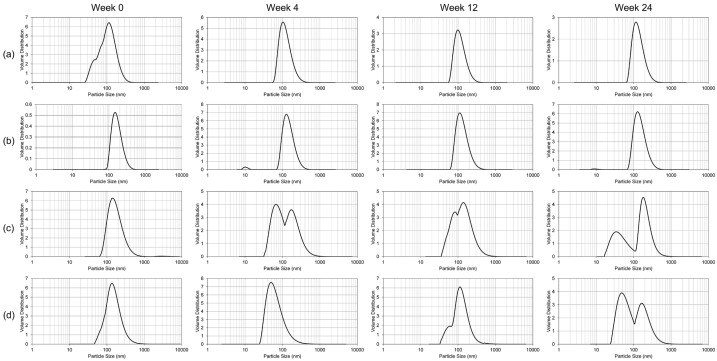
Particle size distribution over time for (**a**) liquid blank NE; (**b**) liquid GT3 NE; (**c**) lyophilized blank NE; and (**d**) lyophilized GT3 NE stored at 4 °C.

**Figure 4:**
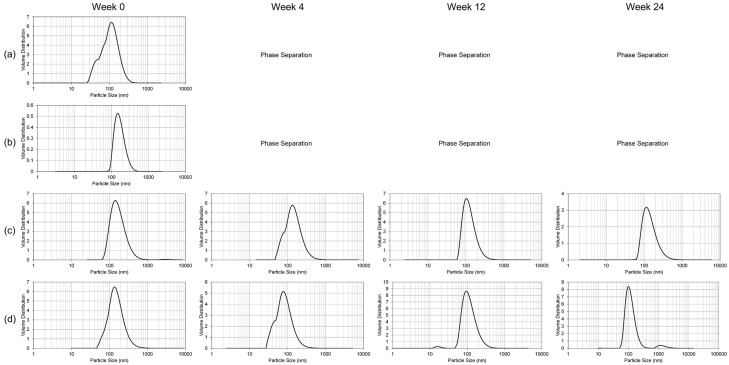
Particle size distribution over time for (**a**) liquid blank NE; (**b**) liquid GT3 NE; (**c**) lyophilized blank NE; and (**d**) lyophilized GT3 NE stored at −20 °C.

The stability of the nanoemulsion formulations was studied over 24 weeks (Figure 2, Figure 3 and Figure 4). At room temperature (25 °C), the liquid NE samples exhibited phase separation between 12 and 24 weeks of storage. No accurate particle size, polydispersity index, or ζ potential measurements could be taken following phase separation. The lyophilized NE samples stored at 25 °C, while visibly indistinguishable in appearance from the initial white, dry powder, began to exhibit inhomogeneity in the particle size distribution starting in the first 4 weeks of storage (Figure 2). The liquid NE samples maintained a consistent size distribution throughout the 24 week monitoring period when stored at 4 °C (Figure 3). In contrast, the lyophilized blank NE stored at 4 °C started to manifest extraneous size distributions within the first 4 weeks, similar to the results at 25 °C, and the lyophilized GT3 NE followed suit within the first 12 weeks. At −20 °C, both the liquid blank NE and GT3 NE had complete phase separation starting from the first measurement time (within 4 weeks), while the lyophilized NE samples maintained a monodispersed state throughout the 24 weeks with the possible exception of the 24 week measurement of the lyophilized GT3 NE (Figure 4).

**Figure 5:**
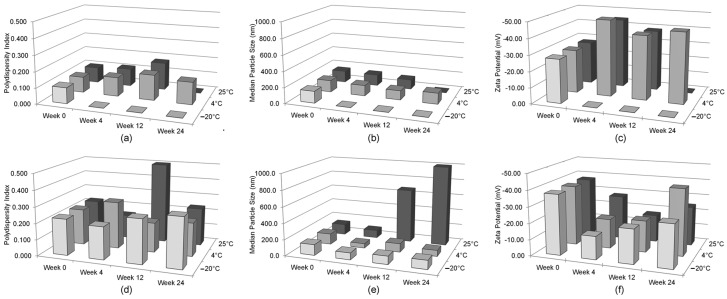
Physical characteristics of the liquid and lyophilized GT3 NE formulations over time. Polydispersity index of (**a**) liquid and (**d**) lyophilized GT3 NE, particle size of (**b**) liquid and (**e**) lyophilized GT3 NE, and ζ potential of (**c**) liquid and (**f**) lyophilized GT3 NE. For those samples which could not be analyzed due to phase separation (e.g., the liquid GT3 NE stored at −20 °C), the measurements are listed as zero on the bar charts. The increase in shades of gray correspond to increase in temperature.

Figure 5 summarizes the change in polydispersity index, median particle size, and ζ potential over 24 weeks. The disparity in polydispersity index measurements between the lyophilized and liquid GT3 NE formulations at day 0 continued throughout the 24 weeks at all storage temperatures (Figure 5a,d). As observed qualitatively for the particle size distribution, the lyophilized GT3 NE at 25 °C had an increased median particle size, most notably after 12 weeks of storage, indicating degradation or instability of the nanoemulsion (Figure 5b,e). The ζ potential measurements of those liquid formulations without phase separation were moderately stable over the 24 week sampling period and were always in excess of −30 mV, but the lyophilized GT3 NE samples did have lower ζ potential measurements during the 24 week sampling period (Figure 5c,f). The increased polydispersity index and reduced ζ potential of the lyophilized GT3 NE at 25 °C indicated that the GT3 NE is not stable in the lyophilized form at room temperature storage; likewise, the liquid GT3 NE at 25 °C and −20 °C were not physically stable at those storage temperatures.

**Figure 6:**
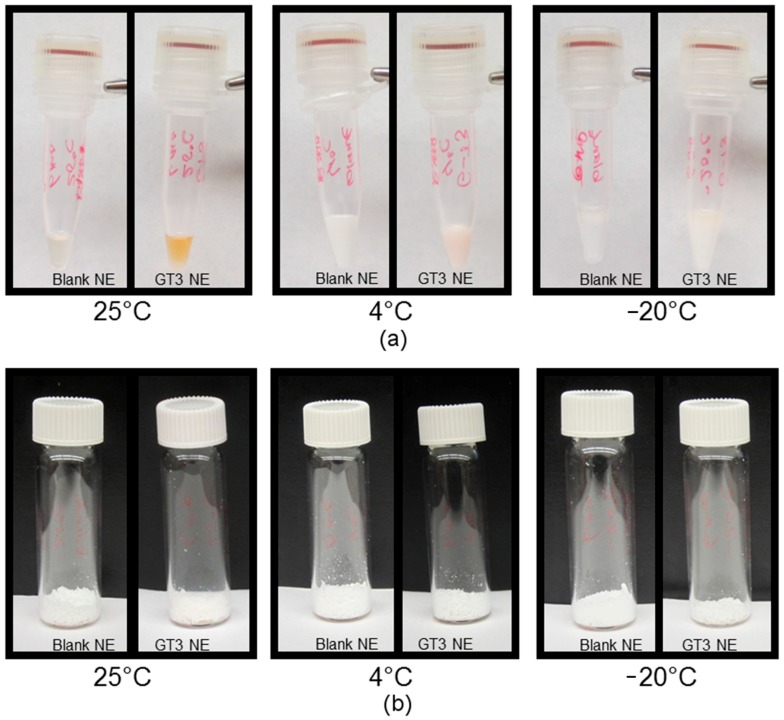
Comparison of (**a**) liquid and (**b**) lyophilized NE with and without GT3 after 24 weeks of storage at 25, 4, and −20 °C.

Although no visual or physical degradation of the nanoemulsion was detected for the duration of the observational period for the liquid GT3 NE at 4 °C and until after 12 weeks at 25 °C, the liquid GT3 NE samples did have discoloration at all temperatures starting from the first monitoring time period (4 weeks). Even the liquid GT3 NE at −20 °C, which was physically separated at 4 weeks, had a pink hue in the oil phase at all time points. The discoloration was, at first, a faint pink discoloration of the liquid GT3 NE samples compared to the white, opaque nature of the liquid blank NE samples. The pink discoloration of the liquid GT3 NE formulations increased in intensity as time passed, and eventually manifested as a reddish/brown color, most notably at 25 °C after 24 weeks of storage (Figure 6). The lyophilized GT3 NE formulations were consistently white in color without any observable degradation at any time during the stability study.

### 2.3. Forced Degradation of γ-Tocotrienol

**Figure 7:**
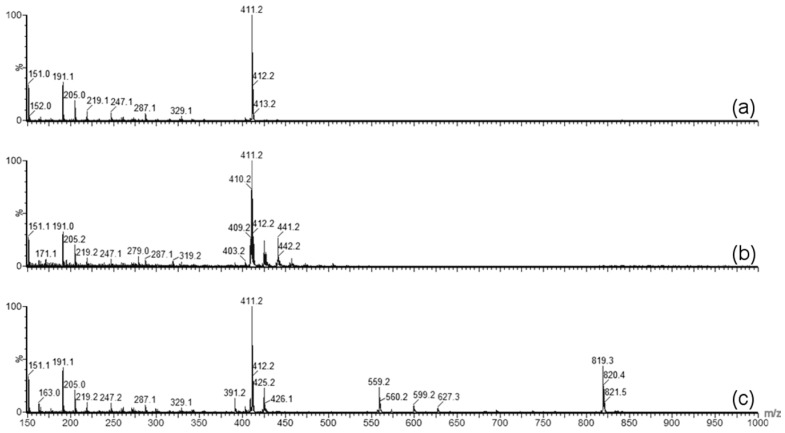
Atmospheric pressure chemical ionization (APCI) mass spectrum of (**a**) GT3; (**b**) peroxide-degraded GT3; and (**c**) heat-degraded GT3. The heat-degraded sample exhibits the presence of GT3 dimer at 819.3 *m/z* and the reddish brown color observed in aged liquid NE samples.

**Figure 8:**
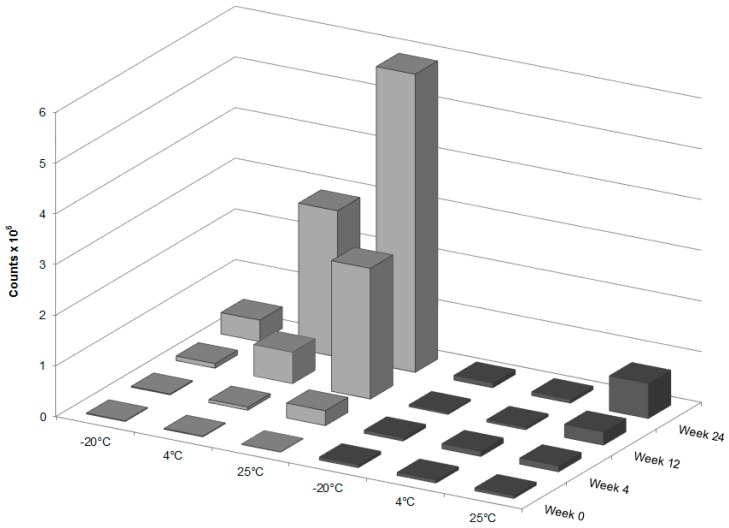
Peak height for the dimer ion located at 819.3 *m*/*z* from the composite mass spectra for the liquid (light gray) and lyophilized (dark gray) GT3 NE samples.

The pink discoloration which developed to reddish/brown over the 24 week storage time, particularly when stored at 25 °C, is a degradation product related to GT3 because the discoloration was only present in those samples containing GT3. The forced degradation of GT3 revealed that the discoloration was related to thermal degradation rather than oxidative stress (Figure 7). Photolytic degradation was not considered because light exposure was minimized during storage. The presence of the discoloration was correlated to the presence of the molecular ion peak at 819.3 *m*/*z*, which is equivalent to twice that of GT3 minus two hydrogens. Thus, this peak likely corresponds to the formation of GT3 dimers in the formulation. The spectrum of the blank NE was subtracted from each GT3 NE spectrum to isolate those molecular ion peaks related to GT3, distinct from those of the nanoemulsion formulation. As in the force degradation study, the presence of GT3 dimers in the samples correlated with degradation and was used as a metric to compare the relative stability of the formulations at different storage temperatures. Figure 8 summarizes the peak height of the dimer ion located at 819.3 *m*/*z* from the composite mass spectra for both the liquid GT3 NE and lyophilized GT3 NE. All of the liquid GT3 NE samples had the formation of dimer ions by week 24 for all storage temperatures. The liquid GT3 NE at −20 °C had the least dimer formation, but the dimer content still increased by 38 times since the start of the trial period. The liquid GT3 NE samples stored at 4 and 25 °C had the most significant dimer content increase with 260 and 530 times more dimer ions at the conclusion of the 24 week study, respectively. Lyophilization considerably reduced GT3 degradation and dimer formation with increases in GT3 dimer of only 1.6, 1.2, and 12.0 times at 24 weeks following storage at −20, 4, and 25 °C, respectively.

### 2.4. Toxicity Study with γ-Tocotrienol Nanoemulsion and Blank Nanoemulsion

**Figure 9:**
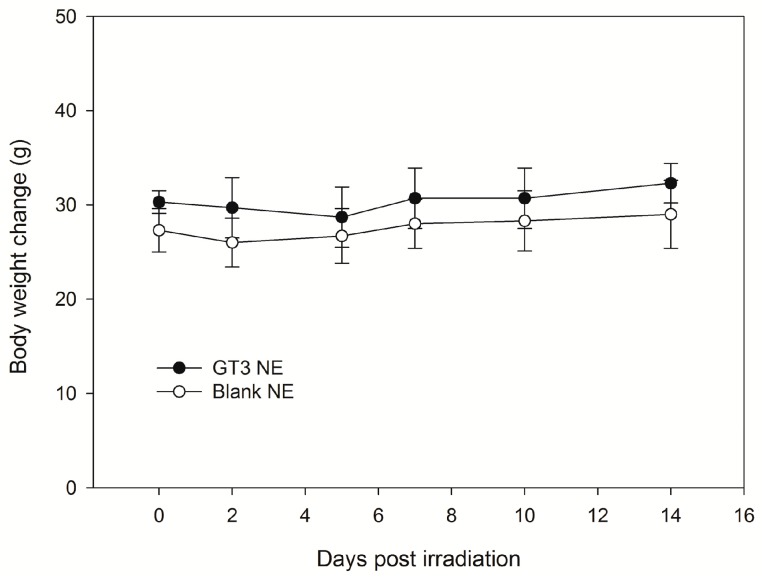
Body weights for CD2F1 mice following administration of NE with and without GT3 (600 mg/kg). Values are presented as mean ± standard deviation, *n* = 3. No significant differences in body weight measurements over time were detected within each treatment group.

A single dose of liquid GT3 NE (600 mg/kg) and blank NE was administered orally to assess the toxicity of the formulated GT3 and the nanoemulsion formulation. The body weight of the mice was monitored over 14 days following dosing, and no significant change in body weight was observed over the experimental time period (Figure 9). Table 1 summarizes the results of the hematological analyses. On day 14, all blood cell counts, neutrophils (NEU), white blood cells (WBC), monocytes (MONO), lymphocytes (LYMP), platelets (PLT), hematocrit (HCT), and red blood cells (RBC) were unchanged following the administration of GT3 NE. Histopathological analysis of the tissues after 14 days resulted in no microscopic evidence of toxicity in any tissues for the GT3 NE and blank NE groups. Several mice exhibited hepatic vacuolar change, but this was considered to be likely clinically insignificant.

### 2.5. Radioprotective Efficacy of γ-Tocotrienol Nanoemulsion

**Figure 10:**
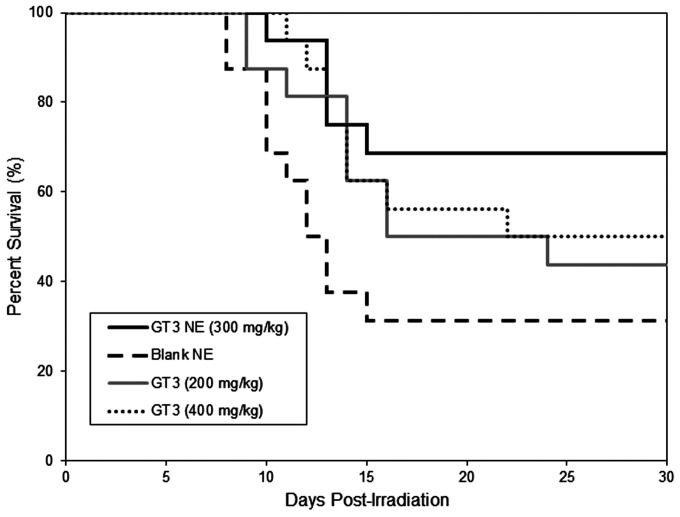
Kaplan–Meier survival curves for CD2F1 mice after irradiation at 9.0 Gy following oral administration of blank NE, GT3 NE (300 mg/kg), and GT3 (200 and 400 mg/kg), (*n* = 16) 24 h prior to irradiation.

Figure 10 illustrates the radioprotective efficacy of mice treated 24 h prior to radiation with 300 mg/kg GT3 of the liquid GT3 NE. The percentages of mice surviving after 30 days for unformulated GT3 at 200 and 400 mg/kg were 43.75% and 50.0%, respectively. The survival rate for those mice dosed with liquid GT3 NE at 300 mg/kg and blank NE were 68.75% and 31.25%, respectively.

## 3. Discussion

The only significant alteration of the nanoemulsion formulation following lyophilization was the polydispersity index, which translates into inhomogeneity in the particle size of the reconstituted lyophilized GT3 NE. While the polydispersity index increased following lyophilization, the median particle size did not significantly change. Thus, lyophilization may impart some instability into the NE following reconstitution. Presence of greater number of large particles can lead to more rapid coalescence or Ostwald ripening. Lyophilized GT3 NE should be functionally the same as the liquid GT3 NE as long as the formulation is utilized within a reasonable time frame following reconstitution.

The note-worthy surface morphology of the lyophilized GT3 NE, with a regular, truncated dome surface, could be aggregates of nanoemulsion droplets which associated or coalesced during the lyophilization process. The bulk of the lyophilized flakes consisted of the cryoprotectant in the formulation, separating and stabilizing the individual nanoemulsion droplets.

Forced degradation was carried out to determine what type of stress caused the degradation of GT3 observed in the stability studies. Photolytic degradation was ruled out as a contributing factor since light exposure was minimized during the stability study, and oxidation degradation and thermal degradation were investigated as possible contributing factors to the degradation of GT3. GT3 and other vitamin E isomers are effective radioprotective agents due to their strong antioxidant capabilities, which makes monitoring of possible oxidative degradation critical to the formulation and storage of GT3. Oxidative degradation of GT3 could reduce its clinical efficacy as a radioprotectant. Additionally, homogenization of the formulation can expose the drug to large liquid/air interfaces, increasing the potential for oxidation of the compound. Therefore, when GT3 degradation was detected in the stability study, identifying the cause of the degradation, and eliminating oxidation, became paramount. The forced oxidation conditions of exposure to peroxide did not reveal degradation of GT3 consistent with the observed degradation in the stability study, both in physical appearance (i.e., discoloration following exposure) and in chemical alteration (i.e., mass spectra similar to the degraded GT3 NE samples). Forced thermal degradation, in contrast, did mimic the degradation pattern observed in the stability study. Specifically, the forced thermal degradation of the GT3 NE corresponded to larger composite mass spectrum peaks located at 819.3 *m/z*, which indicates that the discoloration of the GT3 NE is related, in part, to the formation of GT3 dimers [18]. The formation of GT3 dimers in the formulations is significant because studies have shown a difference in the activity level of GT3 versus GT3 dimer. For example, while GT3 derived from palm oil did inhibit tumor promotion in vitro, the GT3 dimer did not [19]. The improved inter-membrane mobility of tocotrienols compared to tocopherol isomers, and the corresponding faster intestinal transport, has been attributed to the differences in the 16-carbon tail [8,20], which should remain unaffected by dimerization. The chroman ring of the compound, which is the same for each corresponding pair of tocopherol and tocotrienol, carries the active antioxidant group. The antioxidative properties of tocopherols and tocotrienols are influenced by both their chemical reactivity and also by their bioavailability and by the kinetics of their distribution and transport [21]. Whether GT3 dimer is as effective a radioprotectant as pure GT3 requires further investigation.

The optimal storage temperature for the liquid GT3 NE is 4 °C, in order to maintain the properties of the delivery vehicle and to minimize GT3 degradation for 4 weeks of storage. However, storage at 4 °C did not prevent the formation of GT3 dimer ions over time. This result is not surprising considering that the recommended storage temperature for unformulated GT3 is −20 °C. The optimal lyophilized GT3 NE storage temperature was −20 °C—which maintains the structural integrity of both the GT3 and nanoemulsion formulation. Lyophilization is essential to prevent GT3 degradation and to maintain the physical characteristics of the nanoemulsion following thawing and reconstitution. The breakdown of frozen nanoemulsion formulations is a common obstacle for nanoemulsion formulations, and lyophilization of the nanoemulsion prior to storage at freezing temperatures removes this formulation weakness.

There are no drugs approved by the Food and Drug Administration (FDA) to protect first responders deployed in a radiation field for military operations except filgrastim, which has been approved recently for limited use to treat acute radiation syndrome following radiation exposure. Drugs under various stages of development in small and large animals are found to be toxic at effective doses. We reported that subcutaneously (SC) administered δ- and γ-tocotrienol (DT3 and GT3) can prevent pancytopenia and lethality in supralethally irradiated mice, whereas oral GT3 was minimally effective (unpublished data). We have shown here that a single dose of GT3 NE (given orally) was effective to rescue animals from a lethal dose of radiation injury and showed enhanced survival compared to GT3 alone. Both GT3 NE and blank NE proved to be tolerated in the toxicity study following oral delivery at 600 mg/kg. GT3 has already been shown to have no adverse effects in any tissue or organ at a dose of 300 mg/kg following subcutaneous delivery 24 h prior to irradiation [22]. GT3 when delivered subcutaneously can improve post-irradiation survival following total body irradiation at 10.5–12.5 Gy at 400 mg/kg [23]. Formulating GT3 as a nanoemulsion improved the radioprotective efficacy of GT3 over oral delivery of GT3 alone.

## 4. Materials and Methods

### 4.1. Materials

Pure GT3 (95%, High Performance Liquid Chromatography (HPLC)) was purchased from Yasoo Health Inc. (Johnson City, TN, USA) and used to prepare GT3 NE. Unformulated GT3 was prepared by dissolution in 5% Tween-80, 95% saline (supplied to AFRRI by Yasoo Health Inc.). Corn oil, Span-80, Tween-80, lactose, potassium chloride, phosphotungstic acid, methanol, and hydrogen peroxide were obtained from Sigma Aldrich (St. Louis, MO, USA).

### 4.2. Nanoemulsion Preparation

The oil phase of the NE formulation consisted of 30% *w*/*w* GT3 and 70% *w*/*w* corn oil for the nanoemulsion with GT3 and pure corn oil for the blank NE. The surfactant mixture consisted of 1:1 ratio of Span-80 and Tween-80. The composition of the GT3 NE and blank NE was as follows: 32% *w*/*v* oil phase, 8% *w*/*v* surfactant/co-surfactant mixture, and 60% deionized water. The nanoemulsion formulations were homogenized for 5 passes at 20,000 psi on an EmulsiFlex-B3 high pressure homogenizer (Avestin Inc., Ottawa, ON, Canada). The nanoemulsion formulations were diluted with deionized water to a GT3 concentration of 50 mg/mL, filtered with a 0.2 μm cellulose acetate filter (Corning Inc., Corning, NY, USA), and stored at 4 °C until use.

To study the stability of the nanoemulsion formulations in different storage forms and at different storage temperatures, lyophilized samples were prepared for both the GT3 NE and blank NE. The blank NE was prepared using the same composition and process, except this batch does not contain any GT3. Lactose was used as the cryoprotectant for the lyophilized samples. Lactose was added at a 5:1 ratio of lactose to nanoemulsion by adding enough lactose solution. The mixture was flash-frozen in liquid nitrogen prior to lyophilization. The frozen sample was lyophilized at −30 °C for a minimum of 48 h.

### 4.3. Storage

To study the resilience of the nanoemulsion formulations and drug content at different storage temperatures over 24 weeks, three aliquots of the liquid GT3 NE and blank NE, stored in centrifuge tubes, and of the lyophilized GT3 NE and blank NE, distributed in 150–160 mg quantities, were placed at three different temperatures: 25, 4, and −20 °C. All samples at each temperature were light-obscured during the duration of storage. Following 0, 4, 12, and 24 weeks of storage, an aliquot of each sample was removed from each storage temperature and photographed to document the physical appearance of the formulation. Visual observations were recorded of the formulations at each time point, particularly documenting phase separation and discoloration. Additionally, the particle size, ζ potential, morphology, and drug content were analyzed for each sample from each storage temperature.

### 4.4. Particle Size and ζ Potential

The particle size and ζ potential of the formulations were measured with a DelsaNano C particle analyzer (Beckman Coulter Inc., Fullerton, CA, USA). All samples were diluted with deionized water immediately prior to particle size analysis. The lyophilized GT3 NE and blank NE were vortexed for 30 s, and particle size measurements were performed immediately following dilution for both the liquid and lyophilized formulations. The particle size measurements were done using the instrument’s Size Cell option for the particle analyzer. The ζ potential measurements were performed using the instrument’s High Concentration Cell option, and all samples were diluted in 10 mL KCl and vortexed prior to analysis. All measurements were done in triplicate.

### 4.5. Physical Appearance and Morphology

Both the liquid and lyophilized NE formulations were imaged with an S-4800 Scanning Electron Microscope (Hitachi High Technologies America Inc., Gaithersburg, MD, USA). The liquid NE formulations were diluted 50% and stained with 2.5% phosphotungstic acid for 3 min at room temperature. A drop of the negatively-stained NE was added to a formvar-coated copper grid mounted on a dark field stage. The samples were imaged in scanning transmission electron microscopy (STEM) mode at an accelerating voltage of 30 kV. The lyophilized NE formulations were imaged both as a dry powder and after reconstitution with deionized water. The dry NE samples were mounted on aluminum stubs with carbon tape, then sputter-coated (K550X Sputter Coater, Quorum Technologies Ltd., West Sussex, UK) with gold at 2.0 mA for 1 min. The reconstituted lyophilized NE samples were prepared by the same staining procedure as the liquid NE formulations.

### 4.6. γ-Tocotrienol Analysis

The integrity of GT3 incorporated into each formulation was assessed at each time point of the stability study. For the liquid GT3 NE samples, 3.5 µL of the NE was combined with 1 mL of acetonitrile and centrifuged for 5 min at 13,000 rpm (HBI Microcentrifuge; Fisher Scientific, Loughborough, Leicestershire, UK). A 0.5 mL aliquot of the supernatant was diluted with 9.5 mL methanol. For the lyophilized GT3 NE samples, 4.6 mg of each sample was combined with 1 mL of acetonitrile and centrifuged for 5 min at 13,000 rpm. A 0.5 mL aliquot of the supernatant was diluted with 9.5 mL methanol. After dilution of both the liquid and lyophilized GT3 NE samples, all were injected into a single-quadruple, atmospheric pressure chemical ionization (APCI) mass spectrometer (Acquity ultra performance liquid chromatography–mass spectroscopy (UPLC–MS), Waters Corporation, Milford, MA, USA) for analysis. The spectrometer was used in APCI positive mode with a corona voltage of 4.0 kV, cone voltage of 29 V, extractor voltage of 2.0 V, RF lens voltage of 0.6 V, source temperature of 150 °C, APCI probe temperature of 600 °C, cone gas flow rate of 5 L/h, and desolvation gas (nitrogen) flow rate of 500 L/h. The sample feed rate was 20 µL/min, and the spectrum was acquired from 150 to 1000 *m*/*z* for 3 min. The quantification of the suspected presence of GT3 dimer formation was determined by the integration of the mass peak at 819 *m*/*z*.

### 4.7. Forced Degradation of γ-Tocotrienol

Stock solutions of GT3 and GT3 NE were force degraded using peroxide and heat to determine if these degradation methods lead to (1) discoloration as observed after storage of GT3 NE; and/or (2) the formation GT3 dimers. Thermal degradation was achieved by adding 150 µL of a 1 mg/mL stock solution of GT3 in methanol to 10 mL of methanol. This sample and an aliquot of GT3 NE were both placed in an oven at 80 °C for 24 h. For the forced degradation of GT3 with peroxide, two concentrations of peroxide were utilized, 0.3% and 3.0%. At the 0.3% concentration, 150 µL of GT3 stock and 100 µL of 30% hydrogen peroxide solution were combined with 9.75 mL methanol. Sample preparation for forced degradation of GT3 with 3% hydrogen peroxide was done by combining 1 mL of peroxide solution with 150 µL GT3 solution and 8.85 mL methanol. Peroxide degradation of GT3 NE sample was achieved by mixing 1 mL of the GT3 NE with either 10 or 100 µL of 30% peroxide to achieve 0.3% and 30% hydrogen peroxide, respectively. All of the peroxide forced-degradation samples were allowed to stand for 24 h prior to analysis. After storage in the presence of peroxide or heat for 24 h, 100 µL of the degraded GT3 stock and GT3 NE samples were diluted to 1% in methanol for analysis.

### 4.8. Animals

All animal studies were conducted at the AFRRI facility. The protocol (2013-03-004) for these procedures were reviewed and approved by the AFRRI Institutional Animal Care and Use Committee (IACUC) using the principles outlined in the National Research Council’s Guide for the Care and Use of Laboratory Animals. Male CD2F1 mice, 8–9 weeks old (Harlan Laboratories, VA, USA) were maintained at the AFRRI vivarium. The animals were evaluated for microbiological, serological, and histopathological tests by the veterinary staff and determined to be disease- and pathogen-free during the quarantine period. Prior to initiation of experimental procedures, animals were acclimatized for a two-week period. Healthy animals were housed 4 per box in conventional sterile polycarbonate boxes with filter covers (Microisolator, Lab Products Inc., Seaford, DE, USA) and autoclaved hardwood chip bedding. Mice had access to Harlan Teklad Rodent diet 8604 (Purina Mills, St. Louis, MO, USA) and acidified water (pH 2.5–3.0) ad libitum. The animal rooms were maintained at 21 ± 2 °C and 50% ± 10% relative humidity with 10–15 cycles of fresh air hourly and a 12 h/12 h light/dark cycle.

### 4.9. Irradiation

Unanesthetized mice were irradiated bilaterally at AFRRI’s Cobalt-60 γ-irradiation facility. During irradiation, the animals were placed in well-ventilated plexiglass chambers made specifically for mouse irradiation. The mid-line dose to the animals was delivered at a dose rate of 0.6 Gy/min. An alanine/electron spin resonance (ESR) dosimetry system (American Society for Testing and Material Standard E 1607) was used to measure dose rates (to water) in the cores of acrylic mouse phantoms. Phantoms were 3 inches long and 1 inch in diameter, and were located in all empty compartments of the exposure rack. Electron spin resonance signals were measured with a calibration curve based on standard calibration dosimeters provided by the National Institute of Standard and Technology (NIST, Gaithersburg, MD, USA). The accuracy of the calibration curve was verified by inter-comparison with the National Physical Laboratory (NPL) in the UK. The corrections applied to the measured dose rates in phantoms were for decay of the Co-60 source and for a small difference in mass-energy absorption coefficients for water and soft tissue at the Co-60 energy. The radiation field was uniform within ±2% [1].

### 4.10. Drug Administration

GT3 (Tween-80 solution), liquid GT3 NE, and control (blank NE) were given orally at a bolus dose 24 h before radiation.

### 4.11. Single-Dose Acute Toxicity Study

A single dose (600 mg/kg) of GT3 NE or blank NE was given orally (0.2 mL) to male CD2F1 mice (*n* = 6, 12–14 weeks old). All animals were observed constantly for 1 h subsequent to drug administration, then at 4 h, 1 day, and daily for 14 days post-gavage, at which point the study was terminated. Routine cage-side observations were made for signs of pharmacologic and toxicological effects (e.g., tremors, convulsions, salivation, diarrhea, and lethargy), as well as for morbidity and mortality. Each animal was individually marked (ear punch), and body weight was documented shortly before drug administration and then on days 0 (day of oral administration of the drug), 2, 5, 7, 10, and 14. Animals were euthanized on day 14 post-drug administration to evaluate the onset and recovery of treatment-related changes as evaluated through both hematology and the examination of selected tissues for macroscopic and microscopic pathology by a board-certified veterinary pathologist.

### 4.12. Analysis of Blood

Blood (0.6–1.0 mL) was collected from the inferior vena cava (IVC) as a terminal procedure at day 14 from deeply anesthetized animals (Isofluorane; Abbott Laboratories, Chicago, IL, USA). For the hematological analysis, blood was transferred immediately into ethylenediaminetetraacetic acid (EDTA)-containing blood collection tubes and mixed gently in a rotary shaker. Analysis included cell counts for the following: red blood cells (RBC), white blood cells (WBC), platelets (PLT), hemoglobin, hematocrit (HCT), mean corpuscular volume (MCV), mean corpuscular hemoglobin (MCH), mean corpuscular hemoglobin concentration (MCHC), basophils, eosinophils, leukocytes, lymphocytes (LYMPH), monocytes (MONO), and neutrophils (NEU) using an Advia 120-cell counter (Bayer Corporation, Tarrytown, NY, USA).

### 4.13. Gross Pathology and Histopathology

On day 14, a blinded histopathological analysis was performed on all major tissues for signs of toxicity. The tissues analyzed included the following: adrenal glands, aorta (thoracic), bone and bone marrow (sternum), cerebrum, cerebellum, brain stem, pituitary gland, eyes, optic nerves, nasal cavity, larynx, trachea, lungs, heart, tongue, salivary gland, thymus, thyroid gland, stomach, esophagus, duodenum, pancreas, jejunum, ileum, cecum, colon, rectum, liver, gallbladder, spleen, kidneys, haired skin (ventral abdomen and injection site), lymph nodes (mesenteric and submandibular), mammary gland, skeletal muscle, sciatic nerve, and urinary bladder. All tissue samples were fixed in 10% buffered formalin, embedded in paraffin, then sectioned (5–6 µm) and stained with hematoxylin and eosin (H and E; VWR International, Radnor, PA, USA) for routine histopathological processing by a board-certified veterinary pathologist. A severity grade was determined based on the board certified veterinary pathologist’s semi-quantitative assessment of the degree and type of inflammation, necrosis, extent of the lesion, ulceration and adjunct changes to the epidermis, loss or changes to the adnexae and underlying rhabdomyocytes. The grading scheme consisted of ordinal categories as follows: 0 = within normal limits; 1 = minimal—minor changes that involve <10% of region of interest, region of interest (ROI)—; 2 = mild (noticeable changes in the tissue involving 10%–20% of the ROI, with a 10%–20% loss in tissue volume); 3 = moderate (a prominent feature that affects almost half of the tissue, with a 20%–40% decrease in tissue volume); 4 = marked (the lesion is an overwhelming feature that affects most of the ROI, with a 40%–100% decrease in tissue volume); and 5 = severe.

### 4.14. Radioprotection by γ-Tocotrienol Nanoemulsion

Animals (male CD2F1 mice, 12–14 weeks old) were randomly divided into groups of 4 (*n* = 16), and drugs (300 mg/kg GT3 NE, 200 and 400 mg/kg of GT3 Tween-80) or blank NE were administered orally at a bolus dose 24 h before total-body irradiation (TBI) with an estimated LD70/30 dose of γ rays. Irradiated animals were observed three to four times daily, seven days per week for 30 days to monitor survival. Pain and distress was monitored using several criteria including unresponsiveness, abnormal posture, unkempt appearance, immobility, and lack of coordination. A combination of symptoms was used to judge whether an animal had reached a state where euthanasia was indicated, such as the inability of the mouse to right itself, limb paralysis, abdominal breathing, or a constant twitching, trembling, or tremor that lasted for more than 10 s. Animals were euthanized according to American Veterinary Medical Association (AVMA) guidelines.

### 4.15. Statistical Analysis

The differences between mean measurements for the initial liquid and lyophilized GT3 NE were analyzed with a Student’s *t*-test. For a given time point during the stability studies, the statistical comparisons among different samples at different storage temperatures were conducted using ANOVA followed by the Holm–Sidak method for multiple pair-wise comparisons. Differences between two related parameters were considered statistically significant at *p* < 0.05. SigmaPlot (Systat Software Inc., San Jose, CA, USA) software was used for all statistical analyses. For the survival data, Fisher’s exact test was used to compare survival between the two treatments at 30 days, and a log-rank test was used to compare survival curves.

## 5. Conclusions

The formulation of GT3 as a nanoemulsion shows promise for the oral delivery of GT3 as a prophylactic radioprotective agent. To prevent degradation, the GT3 NE must be stored in the lyophilized form at a minimum of 4 °C, preferably at −20 °C. The liquid GT3 NE can be stored at 4 °C, but significant formation of the dimer will occur and accelerate starting between 4 and 12 weeks after preparation. The GT3 NE displayed superior radioprotective efficacy to GT3 alone when delivered 24 h prior to irradiation at 9.0 Gy. Up to 600 mg/kg of GT3 can be safely delivered orally. Further work must be performed to further optimize GT3 NE for oral delivery.

## Figures and Tables

**Table 1:** Results of the complete blood count (CBC) Study.

Parameters Studied	Day 14		
	Reference Values ^1^	Blank NE ^1^	GT3 NE ^1^
RBC (×10^6^ cells/µL)	8.94 ± 0.26	8.73 ± 0.33	8.98 ± 0.11
WBC (×10^3^ cells/µL)	7.59 ± 0.50	2.94 ± 0.57	5.42 ± 0.52
Platelets (×10^5^ cells/µL)	12.11 ± 0.70	14.08 ± 0.01	13.96 ± 1.00
Neutrophils (×10^3^ cells/µL)	0.61 ± 0.07	0.47 ± 0.15	1.19 ± 0.33
Lymphocytes (×10^3^ cells/µL)	5.74 ± 0.27	2.27 ± 0.42	3.86 ± 0.40
Monocytes (×10^3^ cells/µL)	0.10 ± 0.01	0.09 ± 0.02	0.16 ± 0.06
Hematocrit (%)	40.16 ± 1.16	37.27 ± 1.41	38.23 ± 0.33

RBC: red blood cells, WBC: white blood cells. ^1^ Mean ± standard error of the mean (SEM).
